# Effect of Method of Removing Caries-Affected Dentin on the Bond Strength of Composite Resin to Root Canal Dentin

**DOI:** 10.3390/healthcare10112143

**Published:** 2022-10-28

**Authors:** Shiho Otake, Shinya Oishi, Taisuke Ozaki, Masaomi Ikeda, Wataru Komada

**Affiliations:** 1 Department of Masticatory Function and Health Science, Division of Oral Health Sciences, Graduate School of Medical and Dental Sciences, Tokyo Medical and Dental University, 1-5-45 Yushima, Tokyo 113-8549, Japan; 2 Oral Prosthetic Engineering, Graduate School, Tokyo Medical and Dental University (TMDU), 1-5-45 Yushima, Tokyo 113-8549, Japan

**Keywords:** caries-affected dentin, root canal dentin, microtensile bond strength, resin composite, dental diseases

## Abstract

The adhesion of composite resin to caries-affected dentin differs from the adhesion of resin to sound dentin. We evaluated the bond strengths of dual-cure resin composites applied to caries-affected root canal dentin under various clinical conditions and using several caries removal indicators. In the dye stain 1 group, caries were removed to a pale pink stain level using a caries detector. In the dye stain 2 group, caries were removed to a stain-free level using a caries detector. In the probing group, caries were removed to the level of hardness based on probing with a sharp explorer. Additionally, a sound dentin group was used as a control. We compared the resin composite microtensile bond strengths and failure mode distribution among the groups. The bond strengths (MPa) of the probing (64.6 ± 11.9) and the sound dentin (68.7 ± 11.1) groups were significantly higher than those of the dye stain 1 (46.9 ± 7.9) and 2 (47.5 ± 8.4) groups (*p* < 0.05). The removal of caries-affected dentin using a dentin-hardness-based technique showed higher tensile strength than that using a dye stain technique involving removal to any color level. Thus, the caries removal technique used on root canal dentin affects the bond strength of the resin composite.

## 1. Introduction

Fiber-reinforced composite resin posts and cores have been increasingly used for the reconstruction of severely destroyed endodontically treated teeth, instead of cast posts and cores. There are a number of reasons for this. Firstly, the elastic modulus of a fiber post is similar to that of the root dentin structure, and it can reduce the risk of root fracture [1]. Secondly, the favorable light transmission properties of resin composites can produce more esthetically attractive restorations [2]. Lastly, nonmetal restorations using resin composite cores with glass fiber posts eliminate the risk of developing metal allergy reactions [3]. However, clinical evidence that fiber posts reduce the occurrence of root fractures is missing in the recent report [4]. They reported that the incidence rates of root fractures were similar between metal and fiber posts. The adhesion of root canal dentin to composite resin is not yet established.

Accordingly, many studies on fiber posts bonding to root canal dentin have been reported [5,6,7,8,9,10]. In early studies, they reported that composite resins exhibited good bonding to root canal dentin [9,10]. However, in recent studies, the effects of aging on bond strength have been studied. Botter et al. investigated the effect of thermomechanical loading on the bond strength of fiber posts luted with three different resin cements. They suggested a decrease in bond strength after thermocycling. The other reports also suggested that the bond strengths of fiber posts decreased after aging by thermocycling. However, these studies used intact human teeth [5,6,7,8,9,10], while clinical situations often involve root canal dentin of varying qualities and conditions. We need further investigations of root canal dentin bonding in various root conditions, such as caries-affected dentin. For coronal dentin, several reports have been conducted on resin bonding for caries-affected dentin [11,12,13,14,15,16,17]. To obtain caries-affected dentin, artificial caries made in intact extracted human teeth were used in some studies, and caries lesions of extracted human teeth were used in other studies. For caries removal techniques, a laser treatment and a chemomechanical removal technique have been added to the conventional method using burs. The laser treatment decreases the bonding strength of caries-affected dentin to composite resin. Carisolv, as a chemomechanical treatment, did not affect the bonding strength.

Conventionally, the caries removal of root canal dentin has been performed according to the criteria for vital teeth before prosthodontic treatment. That is, we should remove only the caries-infected dentin and leave the caries-affected dentin before bonding procedures for dentin carious lesions because caries-affected dentin in the crown dentin of living teeth can be remineralized. However, caries-affected dentin exhibited lower bond strengths to restorative materials, such as glass ionomer and composite resins, than sound dentin [18,19]. Reportedly, the immediate bond strengths to caries-affected dentin are commonly 20–50% lower than those to sound dentin. Furthermore, the remineralization effect cannot be expected in the case of nonvital endodontically treated teeth. Thus, it is necessary to understand how strongly resin composites will bond to human caries-affected root canal dentin and to develop the criteria for the caries removal of root canal dentin. We have been studying this problem and made a report that aimed to evaluate the influence of caries removal techniques on the bond strengths of dual-cure resin composites applied to caries-affected root canal dentin under various clinical conditions. We showed that caries removal using a dentin-hardness-based removal technique employing a sharp explorer resulted in a higher bond strength than that obtained with a color-based removal technique, where teeth are stained light pink with a caries-detecting solution, which is the standard procedure for the caries removal of vital teeth [20]. In this study, we investigated a different color-based caries removal technique in which caries were removed to a stain-free level, rather than to a light-pink staining level, with caries-detecting solution.

This study aimed to evaluate the bond strengths of dual-cure resin composites applied to caries-affected root canal dentin under various clinical conditions and using several caries removal techniques. Unlike previous studies, we investigated a somewhat different color-based caries removal indicator, in which caries were removed to a stain-free state, rather than to a light staining level, based on the use of a caries detector dye. The null hypothesis was that the group in which caries were removed using a dentin-hardness-based removal technique employing a sharp explorer and the group in which caries were removed to stain-free areas by a color-based removal technique with a caries-detecting solution would have similar bond strength values.

## 2. Materials and Methods

### 2.1. Preparation of Specimens

In this study, we used extracted human teeth: 30 single-root teeth with root caries and 10 single-root teeth without caries. The teeth had been extracted because of periodontal disease, root fractures, or endodontically treated teeth that lost significant coronal tooth structure. After obtaining informed consent from the patients, the teeth were collected under the approval of the ethical review board of Tokyo Medical and Dental University (312/2008). The teeth were stored in normal saline solution at 4 °C.

**Figure 1:**
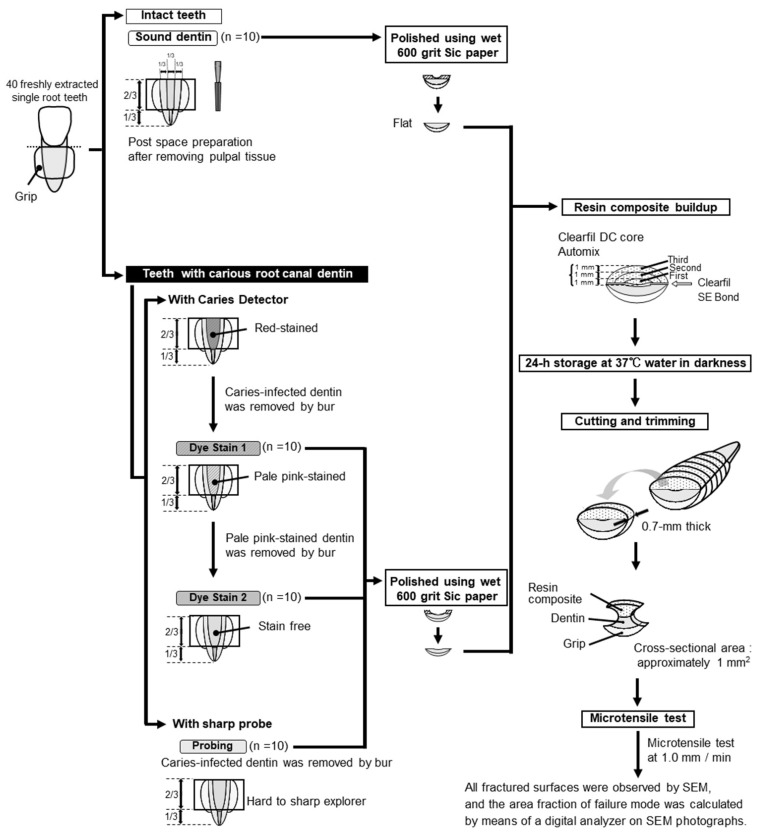
Schematic showing the experimental procedure for microtensile testing.

Figure 1 shows the schematic procedure of this study. To fabricate a grip for the microtensile bond strength test, the external root surfaces were trimmed and built up with a resin composite (Clearfil AP-X, Kuraray Noritake Dental, Tokyo, Japan). The crowns were removed at the cementoenamel junction using a low-speed diamond saw (Isomet, Buehler, IL, USA) under water lubrication. After decoronation, each root was sectioned vertically into halves. The specimens were divided into four groups, as follows.

#### 2.1.1. Dye Stain 1 Group

In the dye stain 1 (DS1) group, the torn surface of the sectioned root was flooded with caries-detecting dye (Caries Detector, Kuraray Medical, Tokyo, Japan) for 10 s and rinsed in water for 5 s to stain the caries lesion [21]. Caries-infected dentin, stained dark-red, was removed using a round bur (ST 1 HP 018, Meisinger, Neuss, Germany). This procedure was repeated until only a pale pink-stained area remained.

#### 2.1.2. Dye Stain 2 Group

In the dye stain 2 (DS2) group, the caries were stained and removed following the same procedure used in the DS1 group, except that the process was continued until the stains were completely removed.

#### 2.1.3. Probing Group

In the probing (PR) group, the caries lesion was detected by scratching the dentin surface with a sharp explorer. Caries-affected dentin was removed with a round bur (STl HP O18, Meisinger, Neuss, Germany). This procedure was repeated until no more scratching dust could be found under the stereoscopic microscope (S300 II binocular telescopic microscope, Inoue attachment, Tokyo, Japan) under 8× magnification.

After removing the caries lesions, a further reduction of specimens was performed with wet 600-grit SiC paper to make the area surrounding the bonding test entirely flat.

#### 2.1.4. Sound Dentin Group

Ten intact single-root teeth were used as sound dentin (SD) specimens (control group).

After decoronation, the pulpal tissue was removed by endodontic files, and the teeth were prepared for root canal using an RTP Reamer (Dentech, Tokyo, Japan). The post spaces were prepared to be one third of the diameter of the root section width and two thirds of the vertical root length [22]. After root canal preparation, each root was sectioned vertically into halves and polished using wet 600-grit SiC paper to ensure that the surface of the bonding area was entirely flat.

### 2.2. SEM Observations

Before completing the priming procedure, the dentin surfaces excavated by the four methods were observed by scanning electron microscopy (SEM).

### 2.3. Microtensile Bond Strength

The root canal dentin surfaces were treated with a bonding agent (Clearfil SE Bond Primer/Bond, Kuraray Noritake Dental, Tokyo, Japan). Before priming, the dentin surfaces were brushed with a microbrush (Shofu, Kyoto, Japan) and rinsed with purified water. After applying SE Primer for 20 s and air drying, SE Bond was applied and light-cured for 20 s. The resin composite (Clearfil DC core Automix, Kuraray Noritake Dental) was built up one layer, which was approximately 1 mm-thick; the procedure was repeated three times (3 mm-thick in total). Each layer was light-cured for 30 s with a light source (Optilux 501, Demetron, CT, USA), and the irradiation direction was set perpendicular to the adhesive surfaces. An accessory radiometer was used to stabilize the power density of the light source to more than 600 mW/cm^2^.

After storage in a 100% humidity incubator at 37 °C for 1 h, the specimens were stored in purified water at 37 °C for 24 h in darkness. The specimens were then sliced into 0.7 mm-thick slabs perpendicular to the bonded surface. A diamond point (AR2f, GC, Tokyo, Japan), mounted in a high-speed turbine handpiece, was used to shape each slab, under water spray, into a dumbbell specimen with a cross-sectional area of approximately 1.0 mm^2^. The dumbbell specimens were glued onto a testing device in a universal testing machine (Autograph AGS-H, Shimadzu, Kyoto, Japan) using cyanoacrylate glue (Zapit, DVA, CA, USA). The samples were then subjected to a tensile force (crosshead speed: 1 mm/min). Ten teeth were used in each testing group, and 3–5 slabs were sectioned from each tooth. The bonding test value for the tooth (*n* = 1) is the average of the measurements of three to five specimens taken from the tooth [23].

The results were analyzed by a one-way analysis of variance (ANOVA), and multiple comparisons were performed by Tukey’s HSD test (*p* < 0.05).

### 2.4. Fracture Mode Analysis

After the bond strength testing, all fractured specimens were submitted to scanning electron microscopy (SEM) observations [24]. The resin and dentin sides of the specimens were mounted on brass tablets and gold-sputter-coated. The fracture modes were examined using an SEM (JSM-5310, JEOL, Tokyo, Japan) to identify the following conditions:

- Cohesive failure in the dentin;
- Failure in the hybrid layer;
- Cohesive failure in the adhesive resin;
- Cohesive failure in the resin composite.

The SEM scans were digitized by scanning and subjecting them to a quantitative image analysis using an image analysis program (ImageJ, National Institutes of Health, MD, USA). In addition, the failure mode distributions were statistically analyzed using a Kruskal–Wallis one-way ANOVA (significance set at *p* < 0.05) and the Mann–Whitney *U* test (significance set at *p* < 0.0083) to compare the differences in the percentage of each failure mode. The statistical *p* value (0.05) was divided according to the number of comparisons among the four groups to avoid the accumulation error of multiple comparisons.

## 3. Results

### 3.1. Microtensile Bond Strength

**Figure 2:**
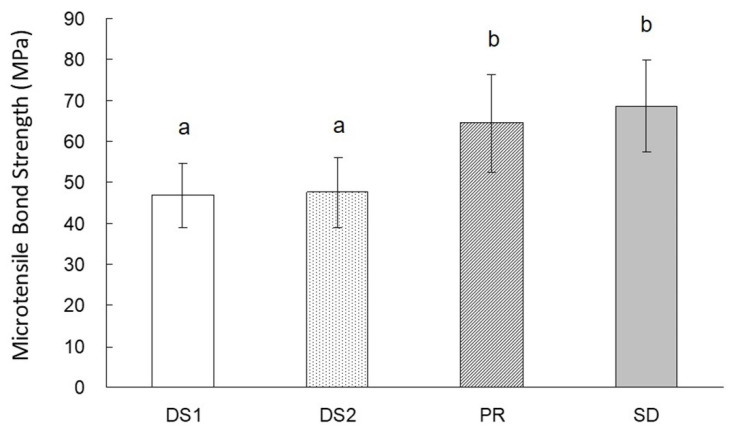
Microtensile bond strength (MPa) to root canal dentin. *n* = 10 in each group, based on Otake 2010. Groups indicated by the same letter are not significantly different (*p* > 0.05).

The results of the microtensile bond strength test are shown in Figure 2. The bonding values of PR and SD were significantly higher than those of DS1 and DS2 (*p* < 0.05) (DS1: 46.9 ± 7.9; DS2: 47.5 ± 8.4; PR: 64.6 ± 11.9; SD: 68.7 ± 11.1 MPa). The bond strength values of DS1 and DS2 were similar, and those of PR and SD were similar.

### 3.2. Fracture Mode

**Figure 3:**
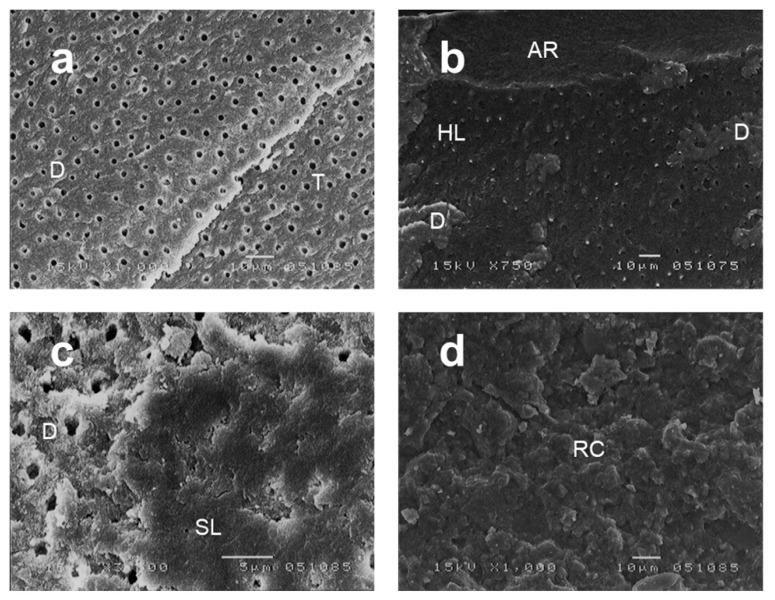
Representative scanning electron microscopy (SEM) images of the fracture interfaces used in the classification of the failure patterns (**a**–**d**) (**a**,**d**: 1000×, b: 750×, c: 3500× magnification), adapted from Otake 2010. (**a**) Failure in the dentin (D) at the fractured surface. Exposed dentinal tubules can be seen. (**b**) SEM image showing three types of failure patterns: cohesive failure in adhesive resin (AR), cohesive failure in dentin (D), and failure in the hybrid layer (HL). (**c**) Fractured surface of DS1 showing fracture in the smear layer (SL). (**d**) Failure in the resin composite (RC) at the fractured surface.

The fracture modes are shown in Table 1, and typical examples of each fracture mode are shown in Figure 3. The Kruskal–Wallis test indicated significant differences in the percentage of surface area affected by cohesive failure in the dentin and cohesive failure in the resin composite (*p* < 0.05). No significant differences were found between the failure in the hybrid layer and the cohesive failure in the adhesive resin. The SD group showed significantly higher values than DS2 and PR in cohesive failure in dentin (*p* < 0.0083). The SD specimens showed significantly lower values in the cohesive failure of the resin composite than the DS2 and PR specimens (*p* < 0.0083).

### 3.3. SEM Observations

**Figure 4:**
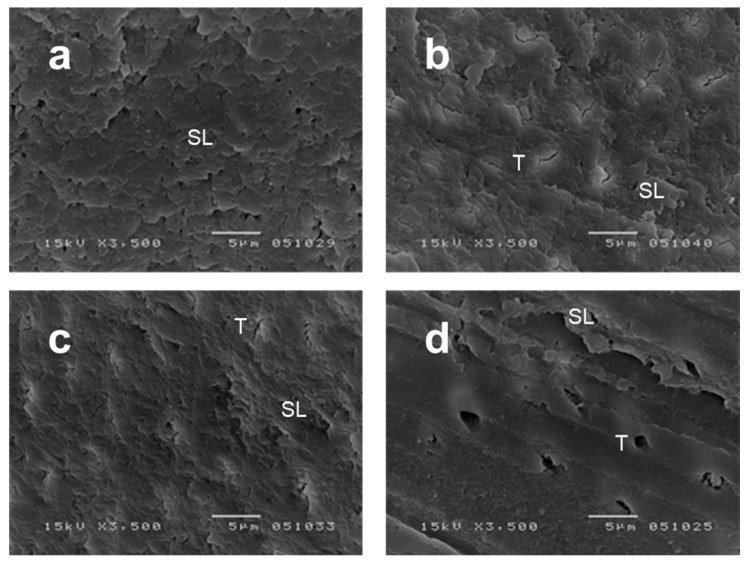
Representative scanning electron microscopy images (3500× magnification) of the dentin surface before priming procedures (**a**–**d**), based on Otake 2010. (**a**) DS1 specimen shows a scraped surface with a rich smear layer (SL). (**b**) DS2 specimen shows a scraped surface with a rich smear layer (SL) and tubule orifice occlusion (T). (**c**) PR specimen showing a thinner smear layer (SL) and tubule orifice occlusion (T). (**d**) SD specimen showing exposed dentinal tubules (T) and a thin smear layer (SL).

Figure 4a–d illustrate the results of SEM observations of the dentin surface after preparation and before primming. Figure 4a,b show similar images of prepared surfaces with a rich smear layers for DS1 and DS2 specimens, respectively. Only the DS2 specimens showed some evidence of tubule orifice occlusion. As seen in Figure 4c, the PR specimens showed thinner smear layers and more occluded tubule orifices than the DS2 specimens. Exposed dentinal tubule orifices and a thin smear layer were observed in the SD specimens, as shown in Figure 4d.

## 4. Discussion

The present study demonstrated that the proper removal of root canal caries is crucial for the success of post and core restorations. Clinically, this process involves the use of the caries removal method, but this approach has not been clearly validated for root canal dentin caries. For coronal caries, only the outer layer of highly infected dentin is excavated [25]. The inner layer of caries-affected dentin can be remineralized and should be preserved. However, caries-affected dentin exhibits lower bond strengths to restorative materials than does sound dentin.

### 4.1. Tensile Bond Strength

It has been shown that the immediate bond strength of a bonding agent to caries-affected dentin is commonly 20–50% lower than that to sound dentin. Nakajima et al. reported that the bond strength to caries-affected dentin was lower than that to intact dentin because of its poor mechanical properties [26]. Accordingly, many studies on fiber posts bonding to root canal dentin have been reported in recent years in terms of pretreatments, reliability, luting cements, aging, etc. [5,6,7,8]. In contrast, caries-affected dentin can be remineralized because calcium phosphate crystals are created inside the dentinal tubules and may promote the growth of apatite [27,28,29,30]. However, in the case of the post and core restoration of endodontically treated teeth, the remineralization of dentin cannot be expected. Firm bonding strength is therefore necessary to retain the post and core restoration to ensure a good long-term prognosis. Thus, clear guidelines for caries removal of root canal dentin, which is different from coronal dentin, are needed. Clinically there are several methods for caries removal. Conventionally, dentin-hardness-based detection, employing a sharp explorer, and a color-based detection, using a caries detector as guide to indicate the proper removal of a carious lesion, have been used. Previous studies have reported on the bonding strength of a resin composite to caries-affected dentin stained with caries-detecting dye (i.e., using a caries detector), but these studies have only examined coronal caries of vital teeth [26,31,32]. Recently laser and chemomechanical treatments have come to be used [11,12,13,14,15,16,17].

The hardness of the caries dentin as detected by a sharp dental explorer or excavator was the most common clinical guide over 40 years ago [33,34]. Recently, a removal device has been developed that can judge the hardness objectively [35]. We reported on the caries removal techniques for caries-affected root canal dentin. We showed that caries removal by the dentin-hardness-based removal technique with a sharp explorer showed higher bond strength than by the color-based removal technique using a caries detector, which is the caries removal standard for vital teeth [20]. This time, we investigated color-based caries removal in a different condition in which caries were removed to a stain-free condition with a caries detector.

Resin adhesives produce lower bond strength to caries-infected and caries-affected dentin than to normal dentin [31]. The results obtained with the DS1 and DS2 specimens denoted the same tendency. A self-etching adhesive system (Clearfil SE Bond) was used in this study because the bonding strength to coronal dentin is well-established [32,36]. Furthermore, the bonding strength to canal wall dentin is reliable with a prolonged photoirradiation time [10]. The bonding values in the PR and SD specimens were significantly higher than those of the DS1 and DS2 specimens. Thus, the null hypothesis—that the removal of caries by a dentin-hardness-based removal technique employing a sharp explorer and the removal of caries to a stain-free level by a color-based removal technique using a caries detector would yield similar bond strength values—was rejected.

### 4.2. SEM Observations

The SEM images revealed that the DS1 and DS2 specimens both had scraped surfaces with rich smear layers. No differences in the bond strength or fracture mode were found between the DS1 and DS2 specimens, while the bond strengths were significantly stronger in the PR and SD specimens. These results suggest that dye-stain conditions (pale pink-stained or stain-free) do not influence the bond strength and that the SE self-etching primer was not strong enough to dissolve the smear layer in the DS1 and DS2 groups. If a smear layer is too thick, it may not be penetrable by a self-etching primer [37]. This implies that the thick smear layers of the DS1 and DS2 specimens, as shown in Figure 4a,b, were too thick to be penetrated by the self-etching primer, which could have resulted in the loss of bond strength. This was ascertained by fracture mode observations, which showed a fracture in the smear layer (see Figure 3c). The PR specimens showed thinner smear layers than those of the DS1 and DS2 specimens. Despite the difference in bonding strength between the DS groups and the PR groups, the fracture mode did not differ among the groups. The thick smear layers in the DS group could cause a loss of strength in the hybrid layer. The SD specimens showed exposed dentinal tubules, thin smear layers, significantly higher bond strengths, and a high percentage of cohesive failure in dentin (Figure 3a). No significant difference was found between the bonding strengths of the PR and SD specimens. The PR specimens showed a high bond strength with cohesive failure in the resin composite, while the SD specimens showed a high bond strength with cohesive failure in the dentin.

### 4.3. Study Limitations and Future Directions

The 30 teeth collected for this study differed in conditions, such as root fracture, residual root stump, with and without gutta percha root canal filling, with metal post, with resin post, with fiber-post-reinforced resin core, and no post preparation. Despite the fact that these conditioned teeth were randomly allocated to the experimental groups, little variability existed among the bonding test data because of the small standard deviation. This suggests that root conditions have little influence on the bonding strength if the caries-affected dentin can be properly removed. However, the situation in this study differed from that in clinical resin composite core build-up because each root was sectioned vertically into halves and tested on a flat plate. In recent years, new adhesive techniques, such as touch cure have been developed, and the bond strength to dentin has been improved [38,39]. However, the results of this study show that it is important to improve the bond strength by ensuring that the condition of the dentin, which is the adherent surface, is appropriate.

The findings of this study suggest that a probing technique that could remove fragile dentin reliably would be effective for the bonding of caries-affected root canal dentin. However, a larger amount of the dentin structure was removed in the PR specimens compared to the DS1 and DS2 specimens. From the standpoint of minimal intervention, it is preferable to remove a smaller amount of tooth structure. The risk of excessive removal of dentin by overstaining has been reported when dyes, such as Caries Detector, were used to remove coronal caries [40]. New caries-detecting dyes prepared with polypropylene glycols, which have a high molecular weight, have been evaluated [41,42]. However, the present study found that the removal of caries-affected dentin, as detected by the caries detector, was not excessive but was insufficient. In a clinical situation, the soft tissue of the root canal dentin is completely removed by the probing technique during post space preparation. The removal of caries by the probing technique ensures the adhesion of the resin composite to the root canal wall dentin. This leads to a better outcome of restored teeth. For caries detection in root canal dentin, new caries-detecting dyes with strong dyeing power should be developed.

## 5. Conclusions

We confirmed that the caries removal technique used for preparing root canal dentin affects the microtensile bond strength. The removal of caries-affected dentin using a dentin-hardness-based technique showed a higher tensile strength than that using a dye stain technique involving removal to any color level. The findings of this study suggest that a probing technique that could remove fragile dentin reliably would be effective for the bonding of caries-affected root canal dentin.

## Figures and Tables

**Table 1:** Percentage of fracture patterns of microtensile specimens.

	Cohesive Failure in Dentin (%)	Failure in Hybrid Layer (%)	Cohesive Failure in Adhesive Resin (%)	Cohesive Failure in Composite Resin (%)
Dye stain 1	57.9 (22.3) ^ab^	12.1 (14.6) ^c^	21.9 (30.6) ^d^	8.1 (9.0) ^ef^
Dye stain 2	47.0 (26.7) ^b^	17.5 (21.1) ^c^	14.1 (13.6) ^d^	21.5 (19.9) ^f^
Probing	38.0 (30.1) ^b^	9.4 (18.5) ^c^	14.4 (17.8) ^d^	38.2 (39.1) ^f^
Sound dentin	80.5 (17.1) ^a^	14.6 (14.1) ^c^	4.0 (4.9) ^d^	0.9 (1.2) ^e^

Values are means (standard deviations), *n* = 10 in each group. Groups identified by different letters are significantly different (*p* < 0.0083).

## Data Availability

Data are available upon reasonable request from the corresponding author.
